# The association of age-related maculopathy susceptibility 2 polymorphisms with phenotype in typical neovascular age-related macular degeneration and polypoidal choroidal vasculopathy

**Published:** 2011-04-20

**Authors:** Hiroaki Bessho, Shigeru Honda, Naoshi Kondo, Akira Negi

**Affiliations:** Department of Surgery, Division of Ophthalmology, Kobe University Graduate School of Medicine, Kobe, Japan

## Abstract

**Purpose:**

To determine the association of age-related maculopathy susceptibility 2 *(ARMS2)* gene polymorphisms with the phenotype of typical neovascular age-related macular degeneration (tAMD) and polypoidal choroidal vasculopathy (PCV) and the effects of photodynamic therapy (PDT).

**Methods:**

The single nucleotide polymorphisms at rs10490924 (A69S) in *ARMS2* of 68 tAMD and 119 PCV patients who underwent PDT were genotyped using the TaqMan assay. The baseline best corrected visual acuity (BCVA) and lesion size were compared among the three genotypes at rs10490924. A multivariate regression analysis was performed to evaluate the influence of the baseline BCVA, greatest linear dimension (GLD), and lesion phenotype (tAMD or PCV) on the association of rs10490924 with the BCVA 12 months after the first PDT.

**Results:**

The mean lesion size was significantly different among the GG, GT, and TT genotypes at rs10490924 in the PCV group, although no significant differences were detected in the tAMD group. PCV patients with a G allele had significantly better vision at 3 months after the initial PDT. tAMD patients with a TT genotype had significantly poorer vision at 12 months after the first PDT. In the multivariate regression analysis, the additive model of the G allele at rs10490924 was associated with a significantly better BCVA 12 months after the first PDT in tAMD and PCV patients.

**Conclusions:**

*ARMS2* variants are likely associated with the phenotype and the effects of PDT in tAMD and PCV.

## Introduction

Age-related macular degeneration (AMD) is a leading cause of central vision loss in the elderly in industrialized countries [1]. The number of patients with AMD has increased remarkably over the years, and further increases in patients with severe visual impairment due to AMD are anticipated [2]. Advanced AMD is clinically classified into atrophic AMD and exudative AMD. Exudative AMD is further sub-classified into typical neovascular AMD (tAMD), polypoidal choroidal vasculopathy (PCV), and retinal angiomatous proliferation [1]. These phenotypes are known to have different characteristics in their natural courses and responses to interventions, such as photodynamic therapy [3,4] and antivascular endothelial growth factor (VEGF) therapy [5], although the reasons are unknown. Recently, several genetic association studies were conducted to explain the different characteristics among the phenotypes of exudative AMD [6,7]and their susceptibility to several interventions, including photodynamic therapy [8]. A report suggested that genetic variants of rs10490924 (A69S) in the age-related maculopathy susceptibility 2 (*ARMS2*) gene were associated with lesion size in PCV cases [9]; however, no replication studies have been reported to date.

PCV is known to have a better response to photodynamic therapy (PDT) than tAMD [3,4], but the reason for this is not understood. Moreover, there is some heterogeneity in the response to PDT among PCV patients [10]. Recent reports have evaluated the association of genetic variants of rs10490924 (A69S) in the *ARMS2* gene with the effects of PDT in neovascular AMD, but a positive association was not found [11,12]. However, a more recent report demonstrated the association of rs10490924 variants with the effects of PDT in Japanese PCV patients [13,14]. They suggested that more studies are needed to confirm their findings.

In this study we investigated the association of rs10490924 (A69S) in *ARMS2* with the phenotypes and visual outcomes of PDT in tAMD and PCV patients in our Japanese cohort.

## Methods

### Study participants

This study was approved by the Institutional Review Board at the Kobe University Graduate School of Medicine and was conducted in accordance with the Declaration of Helsinki. Written informed consent was obtained from all subjects. All cases in this study were Japanese individuals recruited from the Department of Ophthalmology at Kobe University Hospital in Japan.

One hundred and eighty-seven consecutive patients with 68 tAMD (mean age±standard deviation [SD], 76±7 years; ratio of men to women, 53:15) and 119 PCV (mean age±SD, 73±8 years; ratio of men to women, 96:23) who underwent PDT and accepted DNA sampling were retrospectively included in this study. All patients received ophthalmic examinations, including visual acuity measurements, slit-lamp biomicroscopy of the fundi, color fundus photography, optical coherence tomography, fluorescein angiography, and indocyanine green angiography (ICG). The visual acuities were determined using a Landolt C chart and were converted to a logarithm of the minimum angle of resolution for calculations. To classify the patients clearly into tAMD and PCV subgroups, the differential diagnoses were based on ICG [15]. All AMD patients had clear images of choroidal neovascular networks on ICG. The PCV cases showed a choroidal origin of their polypoidal lesions, typically with vascular networks in the posterior poles on ICG and subretinal orange-reddish protrusions corresponding to the polypoidal lesions on ICG. Those patients who received any prior treatment for AMD were not included in this study.

### Photodynamic therapy

All patients in this study were followed up for at least 12 months after their first session of PDT. PDT was performed with standard procedures described previously [16]. The lesion status was assessed every three months, and treatments were performed again when serious retinal detachment, hemorrhage, or macular edema was recognized and accompanied by a leakage on fluorescein angiography (FA) or a defined lesion was observed on ICG. No patients in this study received other treatments or combined therapy during the follow-up period.

### Genotyping

Genomic DNA was extracted from the peripheral blood using QIAmp DNA Blood Maxi Kit (Qiagen, Hilden, Germany). Genotyping was performed using TaqMan^®^ single nucleotide polymorphism (SNP) genotyping assays or Custom TaqMan^®^ SNP genotyping assays (Applied Biosystems, Foster City, CA) on a StepOnePlus™ Real-Time PCR System (Applied Biosystems) in accordance with the supplier’s recommendations.

### Indices compared

The mean age, gender, lesion size (greatest linear dimension [GLD]) based on FA findings and the baseline best corrected visual acuity (BCVA) were compared among the three genotypes of rs10490924 in *ARMS2.* These parameters were measured for each case under masked conditions for genotype. As outcomes of the PDT, the BCVA until 12 months after the initial PDT and the number of PDTs performed during the first 12 months of the treatment term were evaluated.

### Statistical analysis

The parameters were compared among three genotypes using an χ^2^ test for gender and a Kruskal–Wallis test for the indices. For the time-course analysis, two time points in each genotype were compared using a paired *t* test (two tailed). P values<0.05 were considered to be statistically significant.

## Results

The clinical details of the tAMD and PCV patients stratified by the genotypes of rs10490924 in *ARMS2* are listed in Table 1 and Table 2, respectively. Patients with a TT genotype showed a significantly larger mean lesion size than those with GG and GT genotypes in the PCV group. In the tAMD patients, the mean lesion size tended to increase with the number of T alleles, although there was no statistical significance.

**Table 1:** Clinical details of the typical neovascular age-related macular degeneration (tAMD) patients stratified by the genotype of rs10490924 in the age-related maculopathy susceptibility 2 *(ARMS2)* gene.

**n=68**	**G/G**	**G/T**	**T/T**	**P**
Gender (female/male)	0/8	9/25	6/20	0.26^†^
Age (mean±SD, years)	76±5	76±6	76±8	0.87*
GLD (mean±SD, μm)	3063±972	3358±1206	3950±1715	0.37*
Number of PDT sessions/year (mean±SD)	1.6±0.9	1.9±1.0	2.1±1.0	0.57*
Baseline BCVA log MAR (mean±SD)	0.87±0.51	0.76±0.36	0.78±0.42	0.96*
12Mo BCVA log MAR (mean±SD)	0.66±0.40	0.84±0.49	1.03±0.39	0.09*
12Mo-Baseline BCVA log MAR (mean±SD)	−0.21±0.32	0.07±0.51	0.25±0.42	0.01*

Abbreviations: GLD represents greatest linear dimension, PDT represents photodynamic therapy, BCVA represents best-corrected visual acuity, Mo represents month. ^†^ χ^2^ test, * Kruskal–Wallis test.

**Table 2:** Clinical details of polypoidal choroidal vasculopathy (PCV) patients stratified by the genotype of rs10490924 in the age-related maculopathy susceptibility 2 *(ARMS2)* gene.

**n=119**	**G/G**	**G/T**	**T/T**	**P**
Gender (female/male)	3/21	8/38	12/37	0.43^†^
Age (mean±SD, years)	73±9	73±7	72±8	0.88*
GLD (mean±SD, μm)	3475±1615	3051±1550	4342±1737	0.00073*
Number of PDT sessions/year (mean±SD)	1.5±0.7	1.5±0.5	1.6±0.7	0.75*
Baseline BCVA log MAR (mean±SD)	0.79±0.41	0.62±0.32	0.65±0.34	0.27*
12Mo BCVA log MAR (mean±SD)	0.59±0.50	0.47±0.44	0.71±0.41	0.014*
12Mo-Baseline BCVA log MAR (mean±SD)	−0.20±0.39	−0.15±0.45	0.06±0.41	0.012*

Abbreviations: GLD represents greatest linear dimension, PDT represents photodynamic therapy, BCVA represents best-corrected visual acuity, Mo represents month. ^†^ χ^2^ test, * Kruskal–Wallis test.

**Figure 1:**
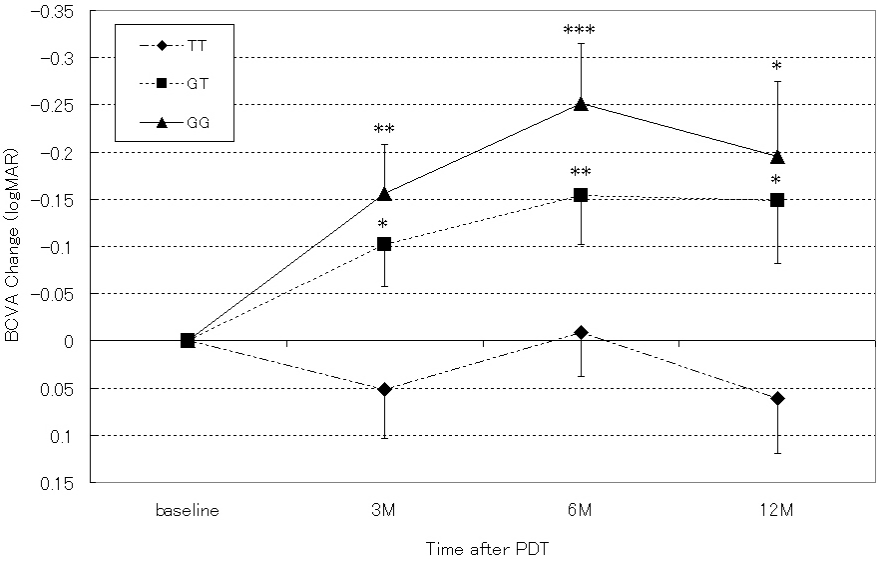
Influence of the genotype at rs10490924 (A69S) in age-related maculopathy susceptibility 2 on the time course of the logMAR baseline best corrected visual acuity (BCVA) value was evaluated in all polypoidal choroidal vasculopathy patients treated by photodynamic therapy (PDT). All values are presented as means±standard error of the mean (SEM). The GG and GT genotypes show significant improvements in their mean BCVA at 3, 6, and 12 months (p<0.05, ** p<0.01, *** p<0.001).

**Figure 2:**
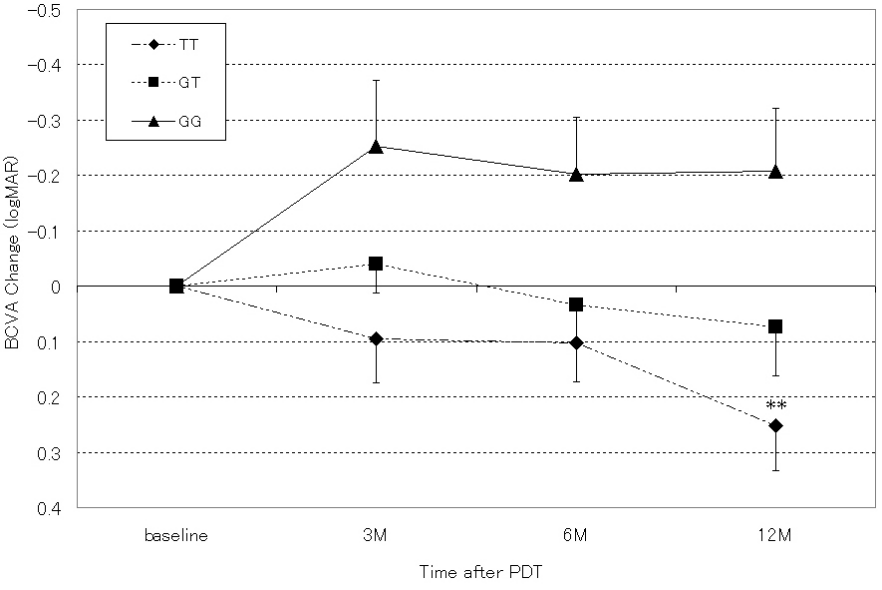
Influence of the genotype at rs10490924 (A69S) in age-related maculopathy susceptibility 2 on the time course of the logMAR best-corrected visual acuity (BCVA) value was evaluated in all typical neovascular age-related macular degeneration (tAMD) patients treated by photodynamic therapy (PDT). All values are presented as means±standard error of the mean (SEM). The TT genotype shows a significant worsening of their mean BCVA at 12 months (** p<0.01).

The mean age, gender, and pretreatment BCVA were not different among the three genotypes in the tAMD and PCV groups. The number of PDT sessions per year tended to increase with the number of T alleles in the tAMD and PCV groups, but no statistical significance was detected. The mean BCVA at 12 months after the first PDT was significantly better for the GG and GT genotypes than the TT genotype in the PCV patients. In the tAMD patients, the mean BCVA tended to be better with an increasing number of G alleles at 12 months after the first PDT, although there was no statistical significance. However, the change of BCVA from the baseline was significantly better for the GG and GT genotypes than the TT genotype in the tAMD and PCV patients. In the time-course analysis, the PCV patients with GG and GT genotypes showed a significant improvement in their mean BCVA at 3, 6, and 12 months post-initial PDT (Figure 1). In contrast, the tAMD patients with a TT genotype showed a significant worsening of their mean BCVA at 12 months post-initial PDT (Figure 2). To evaluate the effects of age, pretreatment BCVA, GLD, PDT frequency, and lesion phenotype (tAMD or PCV), which may influence the effects of PDT [7], we performed stepwise multiple regression analyses with backward elimination methods, including those factors in addition to the number of risk alleles as explanatory variables. The results of this analysis conserved the significance of the association of rs10490924 (A69S) with the improvement of the 12 month BCVA after the first PDT (Table 3).

**Table 3:** Results of the stepwise multiple regression analysis. Prognostic factors for the baseline best-corrected visual acuity (BCVA) at 12 months after photodynamic therapy (PDT) including all phenotypes.

**Prognostic factors**	**SPRC**	**SEM**	**p value**	**95%CI**
Number of risk (T) alleles at rs10490924 in *ARMS2*	0.17	0.041	0.0095	0.027–0.19
Number of PDTs	0.16	0.036	0.0184	0.015–0.16
GLD	0.15	0.00	0.0266	0.000–0.0001
Lesion phenotype (tAMD=0, PCV=1)	−0.21	0.062	0.0017	−0.32–0.075
Baseline BCVA logMAR	−0.4	0.080	<0.0001	−0.65–0.33

Abbreviations: SPRC represents standardized partial regression coefficient, SEM represents standard error of the mean, CI represents Confidence interval, GLD represents greatest linear dimension, BCVA represents best-corrected visual acuity.

## Discussion

We genotyped the well recognized SNP in *ARMS2* in tAMD and PCV patients who underwent PDT and found that the genotype at rs10490924 (A69S) in *ARMS2* was significantly associated with the lesion size in PCV patients and the visual outcome in tAMD and PCV patients at 12 months after their first PDT. Namely, patients with more G alleles at rs10490924 showed better BCVA values until 12 months after the first PDT.

Recent genetic association studies have performed comparative assessments for the association of rs10490924 (A69S) in *ARMS2* among three different phenotypes of exudative AMD: tAMD, PCV [17-20], and retinal angiomatous proliferation [18]. These studies found that the association of rs10490924 (A69S) in *ARMS2* was consistently higher in tAMD patients than in PCV patients, which suggested heterogeneities in the association of this SNP within the AMD phenotype spectrum. The association of *ARMS2* with the lesion size in PCV has been reported previously, and that study emphasized the necessity of replication studies with other cohorts [9]. In the present study we also found an association of *ARMS2* with the lesion size in PCV that supported the previous report. Although a few previous reports from Western countries failed to demonstrate an association of *ARMS2* with the effects of PDT in neovascular AMD [11,12], we found that the coding variants at rs10490924 (A69S) in *ARMS2* were significantly associated with the visual outcome at 12 months post-PDT in Japanese tAMD and PCV patients. This may be explained by differences in the proportion of AMD phenotypes (i.e., neovascular AMD versus PCV) in different ethnic groups or explained by differences in the baseline BCVA between the studies, which may influence the post-PDT BCVA [4]. Our results were consistent with previous reports that suggested a positive association of this SNP with the outcome of PDT in PCV [13,14], a major subtype of Japanese AMD [21]. Although we could not detect a significant association of the genotype at rs10490924 with the lesion size in tAMD patients, there was a tendency for the lesion size to increase with an increasing number of T alleles. We hypothesized that the statistical power was not sufficient to detect a significant association of rs10490924 (A69S) in *ARMS2* with the lesion size of tAMD patients in our cohort and that the association of *ARMS2* may be independent of the AMD phenotype (tAMD or PCV). In fact, the multivariate regression analysis revealed that the number of risk alleles at rs10490924 was an independent factor significantly associated with an improved BCVA at 12 months after PDT. A recent study demonstrated the significant association of SNP rs11200638 in the promoter region of the high-temperature requirement factor A1 (*HTRA1*) gene with the visual outcome after PDT in Japanese exudative AMD patients [8]. Their results were similar to our findings, probably because SNP rs11200638 has a very high linkage disequilibrium with rs10490924 in *ARMS2* [17].

The role of *ARMS2* in PDT is unknown. Recent reports demonstrated that *ARMS2* can affect the lesion size in PCV [9], which may influence the visual outcome at 12 months post-PDT [4]. However, in the present study the results of the multivariate analysis showed that the association of the coding variants in *ARMS2* with the effects of PDT was independent of the pretreatment GLD, BCVA, and lesion phenotype. Kanda et al. reported that ARMS2 distributes to the outer membrane of the mitochondria and may be involved in the regulation of oxidative stress [22]. Reactive oxygen species play a key role by which PDT affects neovascular endothelial cells, followed by thrombosis and the occlusion of neovascular tracts [23]. However, further studies will be needed to elucidate the role of *ARMS2* in the pathogenesis of tAMD and PCV.

The limitation of the present study is the relatively smaller sample size and retrospective nature. A prospective study for the outcome of PDT with a larger population will be needed to disclose further associations of *ARMS2* variants with the effect of PDT in tAMD and PCV patients.

Since PDT is known to induce several gene expression changes in the retina–choroidal complex [23,24], the detailed mechanisms by which multiple genes interact with each other close to the choroidal neovascularization (CNV) is poorly understood. However, the present genetic association study suggests some clinical possibilities that can be applied for personalized therapies in individual tAMD and PCV patients.
